# Decreased plasma lipid levels in a statin-free Danish primary health care cohort between 2001 and 2018

**DOI:** 10.1186/s12944-021-01579-6

**Published:** 2021-10-30

**Authors:** Anna E. Engell, Henrik L. Jørgensen, Bent S. Lind, Anton Pottegård, Christen L. Andersen, John S. Andersen, Margit Kriegbaum, Mia K. Grand, Lise Bathum

**Affiliations:** 1 Department of Clinical Biochemistry, Copenhagen University Hospital Hvidovre, Hvidovre, Denmark; 2 Department of Clinical Medicine, University of Copenhagen, Copenhagen, Denmark; 3 Clinical Pharmacology and Pharmacy, Department of Public health, University of Southern Denmark, Odense, Denmark; 4 Copenhagen Primary Care Laboratory (CopLab) Database, Research Unit for General Practice and Section of General Practice, Department of Public Health, University of Copenhagen, Copenhagen, Denmark; 5 Department of Hematology, Copenhagen University Hospital, Rigshospitalet, Copenhagen, Denmark; 6 Department of Public Health, University of Copenhagen, Research Unit for General Practice and Section of General Practice, Copenhagen, Denmark

**Keywords:** Cholesterol, Lipids, Lipoproteins, Cardiovascular disease, Epidemiology

## Abstract

**Background:**

Lipid levels in blood have decreased considerably during the past decades in the general population partly due to use of statins. This study aims to investigate the trends in lipid levels between 2001 and 2018 in a statin-free population from primary health care, overall and by sex and age.

**Methods:**

In a cohort of 634,119 patients from general practice with no diagnoses or medical treatments that affected lipid levels of total cholesterol (TC; *n* = 1,574,339) between 2001 and 2018 were identified. Similarly, measurements of low-density lipoprotein cholesterol (LDL-C; *n* = 1,302,440), high-density lipoprotein cholesterol (HDL-C; *n* = 1,417,857) and triglycerides (TG; *n* = 1,329,477) were identified.

**Results:**

Mean TC decreased from 5.64 mmol/L (95% CI: 5.63–5.65) in 2001 to 5.17 mmol/L (95% CI: 5.16–5.17) in 2018 while LDL-C decreased from 3.67 mmol/L (95% CI: 3.66–3.68) to 3.04 mmol/L (95% CI: 3.03–3.04). Women aged 70–74 years experienced the largest decreases in TC levels corresponding to a decrease of 0.7 mmol/L. The decrease in LDL-C levels was most pronounced in men ≥85 years with a decrease of 0.9 mmol/L. For both genders, TC and LDL-C levels increased with advancing age until around age 50. After menopause the women had higher TC and LDL-C levels than the men. The median (geometric mean) TG level decreased by 0.4 mmol/L from 2001 to 2008, after which it increased slightly by 0.1 mmol/L until 2018. During life the TG levels of the men were markedly higher than the women’s until around age 65–70. HDL-C levels showed no trend during the study period.

**Conclusions:**

The levels of TC and LDL-C decreased considerably in a statin-free population from primary health care from 2001 to 2018. These decreases were most pronounced in the elderly population and this trend is not decelerating. For TG, levels have started to increase, after an initial decrease.

**Supplementary Information:**

The online version contains supplementary material available at 10.1186/s12944-021-01579-6.

## Background

The lipids cholesterol and triglyceride (TG) are the main lipid components in plasma. They are packed into lipoprotein particles due to the insolubility in water. The four major lipoprotein classes are low density lipoproteins (LDL-C), high density lipoproteins (HDL), very low density lipoproteins (VLDL) and chylomicrons [1]. In the Western world, cholesterol level increases during the first year of life up to 4–5 mmol/L until around age 20, after which levels increases further in both men and women [2]. In the general population, national guidelines recommend total cholesterol (TC) levels < 5 mmol/L, LDL-C < 3 mmol/L, TG < 2 mmol/L and HDL-C > 1 mmol/L [3].

High levels of blood cholesterol with LDL-C as the main cholesterol parameter, are one of the most important risk factors for the development of cardiovascular disease (CVD) and cardiovascular mortality [4–6]. Statins are the most widely used drug for the treating patients with hypercholesterolemia. They inhibit the liver enzyme HMG-CoA reductase competitively, reducing LDL levels, and lowering triglycerides levels [7].

In the past decades, blood cholesterol levels are reported to have decreased, notably in Western Europe and in the USA [8]. The decrease is most likely associated with improved treatment of hypercholesterolemia, especially with statins [9, 10]. Other contributing factors to the decrease might be behavioral and physiological factors, such as increased awareness of risks of hypercholesterolemia and healthier diets, including replacement of saturated fats and trans-fatty acids with unsaturated fat [11, 12] and increased intake of dietary fibers [13]. During the last decades different trends in eating habits have been reported. In Sweden a decrease in reported intake of total and saturated fat until around 2004 has been found. However, a national report analyzing changes in health parameters from 2010 to 2017 concludes, that the group of people, that daily eats an unhealthy diet has increased modestly from 13.3 to 15.9% [14]. Analyzing the trends in cholesterol levels in populations is important for understanding the dynamics of CVD, as CVD is still the leading cause of mortality and morbidity in Western countries [12, 15, 16]. A dramatic decline in the frequency and mortality from CVD, especially ischemic heart disease (IHD) has taken place in many Western countries. Probably due to improved treatment and changes in CVD risk factors [17].

Previous studies have examined changes in lipid levels in hypothetical statin-free populations, where the impact of statins on lipid levels was based on estimations [8, 18]. Therefore, the main aim of this register-based cohort study was, to quantify the temporal trends in TC, LDL-C, HDL-C and TG levels in a population of statin-free patients from general practice in Denmark between 2001 and 2018.

## Methods

### Study population and data sources

The present study includes data from primary health care patients in the Copenhagen area of Denmark. All Danish residents have free and direct access to general practitioners (GPs), who can refer patients to blood testing. In this study, patients who were referred to blood testing for the analysis of TC, LDL-C, HDL-C or TG from 2001 through 2018 from the Copenhagen Municipality and the former Copenhagen County were included.

The test results were retrieved from the Copenhagen Primary Care Laboratory (CopLab) database (2001 through 2015) [19] and the laboratory information system Labka (from 2016), which includes blood test results from primary health care patients.

Individual-level data on residence in Denmark and deaths were retrieved through registries at Statistics Denmark. Data concerning drug use and diagnoses were retrieved from the Danish National Prescription Registry [20] and the Danish National Patient Registry [21]. Data linkage was performed using the unique Danish Civil Registration Number assigned to all residents in Denmark [22].

**Fig. 1:**
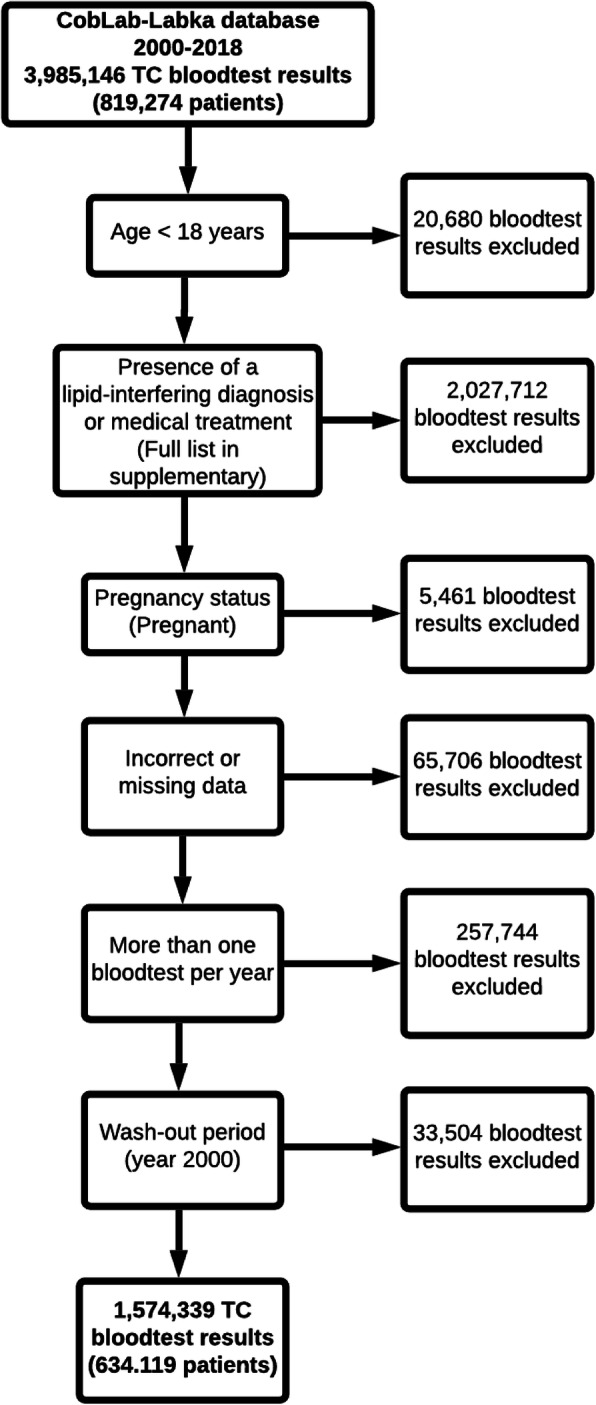
Flowchart of the patient exclusion process

To identify adult patients whose lipid levels were not influenced by external factors affecting the lipid metabolism, different exclusion criteria were applied (Fig. 1). Specifically, patients with diagnoses or a medical treatment potentially affecting the lipid levels present during a three-year period before a given blood test were excluded. Medical treatment was defined as a minimum of two redeemed prescriptions. A full list of diagnoses and medical treatments causing exclusion can be found in the supplementary material. None of these drugs are sold as over-the-counter drugs. Women, who were pregnant at the time of the blood test, were also excluded, as were patients under the age of 18 years. Furthermore, patients with missing or invalid data were excluded (for instance non-numeric blood test results, immigration after, or emigration before the blood test). Finally, only the first measurement for each calendar year was included, if the patient had more than one lipoprotein blood test result per year.

### Measurements

Different laboratories have carried out the analysis of lipid levels during the study period. Until 2016, the Elective Laboratory of the Capital Region analyzed all tests from GPs in the Capital Region. From 2016, the hospital laboratories analyzed all blood tests in the Region. Data from three of the Region’s laboratories were used in this study. All measurements were based on colorimetric methods applied to different analyzers and the laboratories participated in international external quality control schemes to confirm the reliability of the assays (Supplementary Fig. 1). LDL-C was routinely estimated from the Friedewald equation [23]: LDL-C = TC – HDL-C – (0.45 * TG). LDL-C was not estimated if TG was above 4.5 mmol/L. During the study period, it was shown that lipids change only minimally in response to food intake [24, 25], therefore TG and LDL-C were only obtained in the fasting state until the end of 2009. After this, TG and LDL-C measurements are a mixture of fasting and non-fasting test results.

### Statistical analyses

The distribution of the concentration of TC, LDL-C and HDL-C levels were found to be normally distributed, therefore the mean was used in further calculations. TG levels were log normally distributed; therefore, the median (geometric mean) was used.

In the main analysis, the mean (or median) level of TC, LDL-C, HDL-C and TG for each calendar year from 2001 to 2018 were calculated. If a visual trend was present in the lipid levels in the main analysis, a secondary sex and age specific analysis was performed. Sex and age specific lifetime trends in mean (or median) lipid levels including 95% CI for the first year of the study period (2001) and the last (2018) were estimated. Patients were categorized in 5-year age groups (18–24, 25–29, 30–34 … 70–74, 75-,79, ≥ 85 years). In all analyses, the level of statistical significance was set at *p* < 0.05. Analyses were performed using SAS 9.4. (SAS Institute Inc., Cary, North Carolina).

### Sensitivity analysis

To assess the potential impact of the sampling of the study population, the analysis of lifetime trends in TC levels in two other study populations was performed. First, in a study population, where all lipid blood test results from 2001 to 2018 were included, for example, if a patient had multiple tests per year, all results were included in the analysis. Secondly, the analysis in a study population only including the first ever TC blood test result during the whole study period was carried out.

### Supplementary analyses

Additionally, the longitudinal trends in the lipid CVD biomarkers; small dense low-density lipoprotein-cholesterol (sdLDL-C) [26] and Non-HDL levels from were estimated. Lastly, mean LDL-C levels from 2001 to 2018 using the Sampson equation [27] were calculated to examine the impact of the equation used for LDL-C levels during the study period (Friedewald).

## Results

The study population included TC results from 634,119 patients (1,574,339 blood tests), LDL-C results from 559,889 patients (1,302,440 blood tests) patients, as well as HDL-C and TG results from 590,173 (1,417,857) and 572,215 (1,329,477) patients respectively. The characteristics and mean TC levels in the included and excluded population were compared (Table 1)*.* The two groups did not differ concerning sex and age. However, TC levels were lower, and the frequency of blood testing was higher in the excluded population.

**Table 1:** Characteristics of the included and excluded population

	Included	Excluded	***P***-value
*n* (individual patients)	634,119^a^	185,155	
Sex (women/men), n (%)	333,659 (52.6) / 300,460 (47.4)	97,679 (52.8) / 87,476 (47.2)	0.3
Age (years), mean (SD)	49.0 (17.4)	48.9 (17.3)	0.5
Mean number of TC measurements	2.4 (2.1)	5.1 (6.4)	
TC, mean (SD)	5.4 (1.1)	5.0 (1.2)	< 0.0001
LDL-C, mean (SD)	3.3 (0.9)	2.9 (1.0)	< 0.0001
HDL-C, mean (SD)	1.5 (0,4)	1.5 (0.5)	< 0.0001
TG, mean (SD)	1.5 (1.1)	1.7 (1.4)	< 0.0001

Data are expressed as n (%) or mean (mmol/L) ± SD. *TC* total cholesterol, *LDL-C* low-density lipoprotein cholesterol, *HDL-C* high-density lipoprotein cholesterol, *TG* Triglyceride

^a^Number of patients with at least one included TC blood test result between 2001 and 2018

### Trends in lipid levels and frequency of TC blood testing from 2001 to 2018

**Fig. 2:**
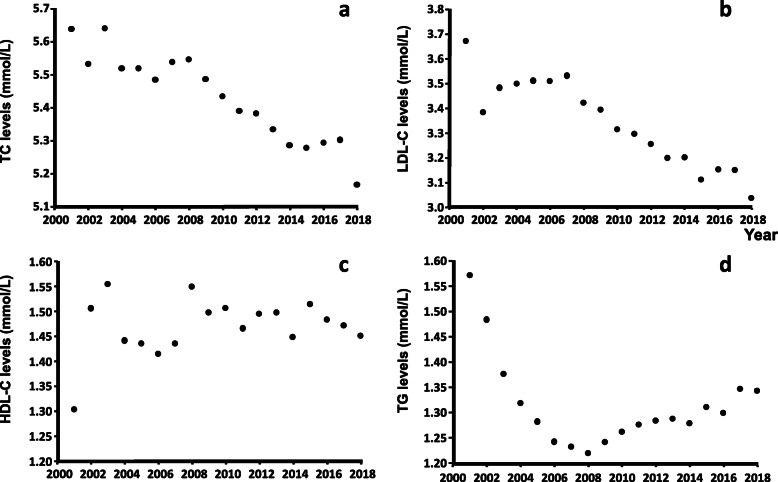
Annual trends in TC (a), LDL-C (b), HDL-C (c) and TG (median values) (d) levels in mmol/L from 2001 to 2018. Data are presented in mean values (mmol/L). Estimates are based on the first result each year, if multiple results per patient per year were available

The mean TC level decreased from 5.64 mmol/L (95% CI: 5.63–5.65) in 2001 to 5.17 mmol/L (95% CI: 5.16–5.17) in 2018, corresponding to an 8% decrease over the 17-year period (Fig. 2). The mean LDL-C level decreased by 17% from 3.67 mmol/L (95% CI: 3.66–3.68) to 3.04 mmol/L (95% CI: 3.03–3.04). For HDL-C, no specific trend was observed in the observation period, while TG levels decreased from 2001 until 2008 by 0.35 mmol/L, after which it increased slightly (Fig. 2).

The frequency of TC blood tests increased considerably during the study period, from 52,533 (45,061 individual patients) TC blood tests in 2001 to 127,767 (109,772 individual patients) TC blood tests in 2018. Similar increases were present for the other lipids (data not shown).

### Lifetime trends in lipid levels in 2001 and 2018 according to sex and age

**Fig. 3:**
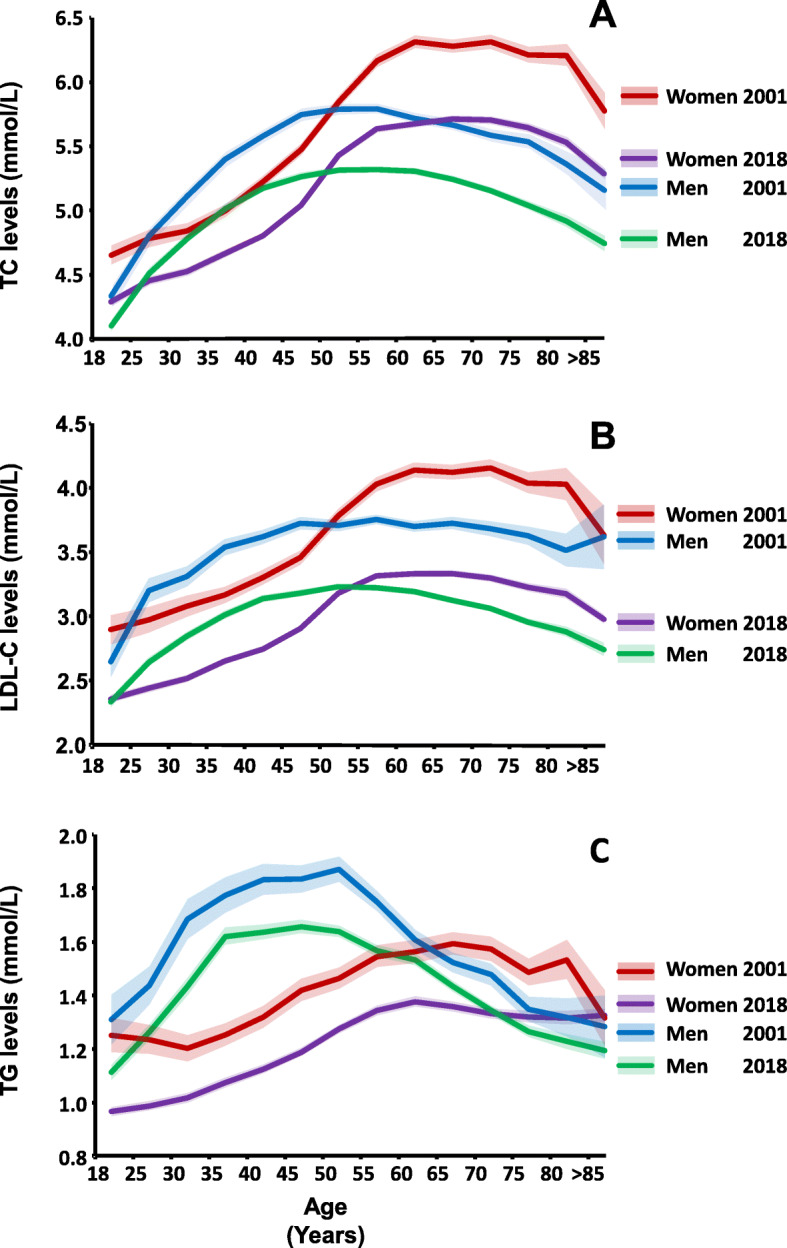
Sex and age related lipid levels (mmol/L) in 2001 and 2018 for TC (a), LDL-C (b) and median levels of TG (c). Estimates are based on the first result each year, if multiple results per patient per year were available. TC cohort size in 2001: 45,061 patients (19,714 women/25,347 men). TC cohort size in 2018: 109,772 patients (55,758 women/54,014 men)

Women had lower TC levels compared to men, until the age of 50 (except the youngest age group), corresponding to the time of menopause, after which they had higher levels than men (Fig. 3a). For both men and women, TC levels decreased with advancing age in the oldest populations. A decrease from 2001 to 2018 in TC levels was also observed in this analysis varying among the age groups. For women, the 80–84 years old experienced the largest decrease in mean TC levels of 0.7 mmol/L from 2001 to 2018 (11%), while the 30–34 years old experienced the smallest decrease of 0.3 mmol/L. Among men, the decrease ranged from 0.2–0.5 mmol/L, with men aged 75–79 experiencing the largest decrease from 2001 to 2018 (9%).

As for TC, the mean LDL-C levels were also lower in women until around menopause, after which they had the highest levels (Fig. 3b). A decrease in mean LDL-C levels from 2001 to 2018 was observed in all age groups. In women, the decrease ranged from 0.5 mmol/L in the 35–39 years old to 0.9 mmol/L in the 70–74 years old, corresponding to a maximum decrease of 19%. Among men, the decrease ranged from 0.3 mmol/L in the youngest age group to 0.9 mmol/L in the oldest age group, corresponding to a decrease of 24%. The TG levels of women increased slightly with advancing age until the age of 70 years after which they decreased. In men, TG levels peaked in the middle-aged age groups and decreased with advancing age thereafter (Fig. 3c). Among women, the decrease in median TG levels from 2001 to 2018 remained unchanged with an average decrease of approximately 0.2 mmol/L in all age groups except the oldest age group. The corresponding decrease for men was also stable at approximately 0.2 mmol/L until about age 55–59 years, where the difference between median values in 2001 and 2018 evened more out.

No sex and age specific analysis was carried out for HDL-C, since the levels did not show an evident trend between 2001 and 2018.

### Sensitivity analysis

When including all TC blood test results or when restricting to first TC blood test ever, virtually unchanged estimates were obtained (Supplementary Fig. 2).

### Supplementary analyses

The annual mean levels of non-HDL-C (Supplementary Fig. 3a) and sdLDL-C (Supplementary Fig. 3b) decreased from 2001 to 2018 corresponding to the decrease found in the main analysis. LDL-C mean levels from 2001 to 2018, calculated with the Sampson equation [26], showed virtually the same trend compared to LDL mean levels calculated with Friedewald equation (Supplementary Fig. 3c).

## Discussion

In this register-based cohort study including a statin-free primary health care population, considerable changes in lipid levels during the past 18 years were observed. Overall, the mean TC, LDL-C and median TG levels decreased markedly from 2001 to 2018 regardless of sex and age. The decrease in both TC and LDL-C seems to continue, whereas the decrease in TG shows an increasing tendency from 2008. No specific trend was observed for HDL-C. Supplementary analyses of the annual trend in non-HDL-C and sdLDL levels showed a similar decreasing trend.

### Comparisons with other countries

The results are supported by prior studies, where the populations use statins, also reporting a decline in lipid levels [8, 16, 18, 28–31]. For example, a decrease in TC from 6.2 mmol/L to 5.5 mmol/L (11%) between 1994 and 2014 in a Northern Swedish population was reported by the WHO MONICA study, which included 8895 individuals. Similar to this study, the oldest patients experienced the largest decrease, but they also had the highest levels at baseline [8]. It has been estimated that statin treatment accounts for one third of the decline [8, 18]. Another study found a decrease in TC from 6.3 mmol/L to 5.0 mmol/L (21%) during a period from 1986 to 2002 in a Northern Swedish population. After 2002 the TC levels tended to increase until 2010, where the observation period ended [31]. A decrease of 13% in LDL-C levels from 2001 to 2008 was found in an American population of 105 million patients with no exclusions due to medical treatment. The magnitude of the decrease was largest in the oldest patients in this study as well. After 2008, the decrease stabilized until 2011 [30]. No trend towards a stabilization or an increase in cholesterol levels as reported in some studies [30, 31] was found.

The changes in TC levels according to sex and age are similar to the findings of recent large cohort studies Dutch and South Korean populations [32, 33]. Especially TC and LDL levels show sex specific trends. Several studies report that the menopause transition is associated with changes in lipid levels [34, 35]. Postmenopausal women have higher levels of TC, LDL-C, TG and HDL-C, than premenopausal women. The mechanism can be explained by the complex hormonal effects of estrogens on lipid metabolism [36]. Clinically, the menopause-associated changes in lipid levels may play an important role in women’s increased risk of CVD in the post-menopausal years [35].

### Strengths and limitations

In contrast to prior studies, this study is based on results from a statin-free study population, preventing statin treatment from influencing the estimates of the decrease in lipid levels. The study population in this study is comprised of primary health care patients. By using results from a clinical setting, the reported trends in lipid levels reflect lipid changes in real patients. The majority of studies describing the changes in cholesterol levels rely on results from volunteers participating in population surveys, which increases the risk of participation bias. If only the most resourceful and healthy individuals participate in the survey [37], lipid levels could potentially be underestimated.

The frequency of TC blood testing increased considerably during the observation period. This increase is also reported in comparable European populations [38]. Therefore, part of the decrease could be explained by more healthy patients having their cholesterol levels measured compared to 20 years ago. If so, the magnitude of the decrease could be overestimated, due to the greater portion of normal results. However, the result of the sensitivity analysis, when restricting to first TC blood test ever, showed a similar decreasing trend in lipid levels. The number of patients in this analysis are decreasing annually, since fewer patients have their first TC blood test taken during the study period. Therefore, the impact of the increase in blood test frequency cannot solely explain the observed decrease in TC (and LDL-C) levels.

A dramatic decline in mortality from ischemic heart disease of 81% from 1990 to 2015 in Denmark have been reported [17]. This decline is both attributed to the use of cholesterol lowering treatment, as well as improved treatment regimens [17]. In addition, the findings of this study of a decrease in lipid levels in a statin-free population could also be a contributing factor to the decline in ischemic heart disease. However, some studies have associated low lipid levels with increased all-cause mortality in a population without CVD and diabetes among the oldest patients [39].

Unfortunately, we did not have access to health-related individual information e.g. about BMI, level of daily physical activity, diet, alcohol consumption and smoking status. Data from other studies indicate that there has been a decrease in the number of Danes smoking and drinking in this period [14]. However, data also indicate that the diet nationally has changed to an unhealthier diet in the same period [14] and that the proportion of overweight or obese people in Denmark have increase from 46.8% in 2010 to 51.0% in 2017 [14]. Consequently, national changes in these health markers from 2010 to 2017 are divergent, and cannot solely explain the decrease in lipid levels [14].

TG levels decreased initially before increasing again. The shift from only accepting fasting TG blood tests to a mixture and fasting and mostly non-fasting blood tests in 2009 following alterations in clinical recommendations [25] could explain this increasing trend. Further, this could have affected LDL-C values estimated using TG values with the Friedewald equation. However, the decreasing trend in LDL-C levels remained unchanged when calculated with the Sampson equation [26]. Additionally, there is clinical evidence from several large population studies that lipid levels change only minimally in response to normal food intake [24, 40, 41]. Therefore, the impact of the shift from accepting fasting to non-fasting blood tests in 2009 must be limited. Since increased non-fasting TG levels recently have been found to be a marker of elevated CVD risk and total mortality, it is still important to continuously monitor TG levels [42].

In the diagnostic process of hypercholesterolemia, both desirable concentration limits as well as health related reference intervals are used internationally. Based on the results of this study showing a considerable decline in cholesterol levels, attention must be drawn to the accuracy of the reference intervals and updating those if outdated. In Scandinavia the current reference intervals are from 2004 [43]. The results from this study could indicate that these do not contain the central 95% of the population anymore, therefore establishment of updated reference intervals could be considered.

In this study, the trends in lipid levels in relation to sex and age were estimated, but the underlying causes of the decrease in cholesterol levels are beyond the scope of this study. Likely, they are associated to several changes in behavior related to health. Therefore, future studies are needed to further understand the magnitude and distribution of the decrease in cholesterol levels in different populations. For example, factors such as socioeconomic status and health risk profile might have differentially affected the adaption of the changes leading to decreased levels of cholesterol.

## Conclusions

Elevated cholesterol levels are an important risk factor of CVD. Monitoring of the longitudinal changes in cholesterol levels in the population is therefore important. This study of over 600,000 primary health care patients free from statins reports a considerable decline in TC and LDL-C levels during the 2001 to 2018 period. The older patient groups experienced the largest decrease corresponding to a decrease in TC levels of 11% in the women aged 70–74 years and a decrease of 24% in LDL-C levels in men aged ≥85 years. These decreases do not seem to decelerate at the end of the observation period. However, TG levels have increased slightly after and an initial decrease.

The trends in lipid levels during a lifetime show that the women’s TC and LDL-C levels exceed the men’s after menopause after the age of 65–70 for TG.

## Supplementary Information

**Additional file 1: Figure 1.** Mean deviation of TC from target value set by the external quality assessment organization from 2003 to 2018. Values are presented as deviation in percent with 95% CI. Data from 2001 to 2002 were not available.

**Additional file 2: Figure 2.** Sex and age related mean TC levels in 2001 and 2018 in a cohort restricting to first TC blood test ever (a), and including all blood test results regardless of multiple results per year (b).

**Additional file 3: Figure 3.** Annual trends in Non-HDL-C (a), sdLDL-C (b) levels from 2001 to 2018, and LDL-C calculated with the Sampson equation (c). Data are presented in mean values (mmol/L). Estimates are based on the first result each year, if multiple results per patient per year were available.

## Data Availability

Access to statistical codes on which the conclusions of the paper rely, will be shared on reasonable request to the corresponding author.

## Abbreviations

CVD: Cardiovascular disease
HDL-C: High-density lipoprotein cholesterol
LDL-C: Low-density lipoprotein cholesterol
sdLDL-C: SMALL Dense Low-Density Lipoprotein-Cholesterol
TC: Total cholesterol
TG: Triglyceride
