# Supplementary figures and images for: Decreased plasma lipid levels in a statin-free Danish primary health care cohort between 2001 and 2018
Source: Lipids Health Dis. 2021 Oct 30;20:147. doi: 10.1186/s12944-021-01579-6 (PMC8557491; doi:10.1186/s12944-021-01579-6)

## Slide 1
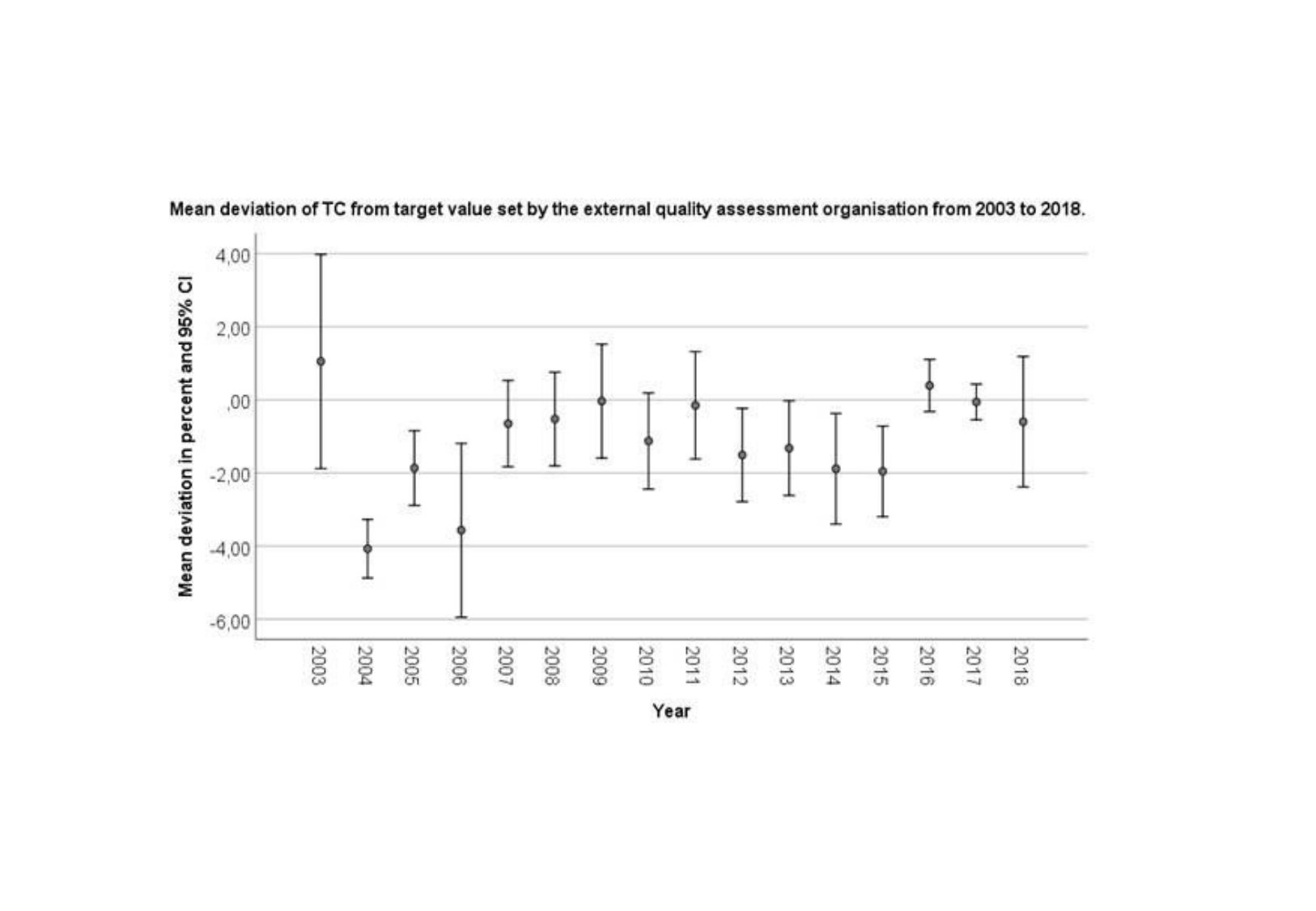

Supplement: Supplementary file 1 — Additional file 1: Figure 1. Mean deviation of TC from target value set by the external quality assessment organization from 2003 to 2018. Values are presented as deviation in percent with 95% CI. Data from 2001 to 2002 were not available. [file 12944_2021_1579_MOESM1_ESM.pptx]

## Slide 1
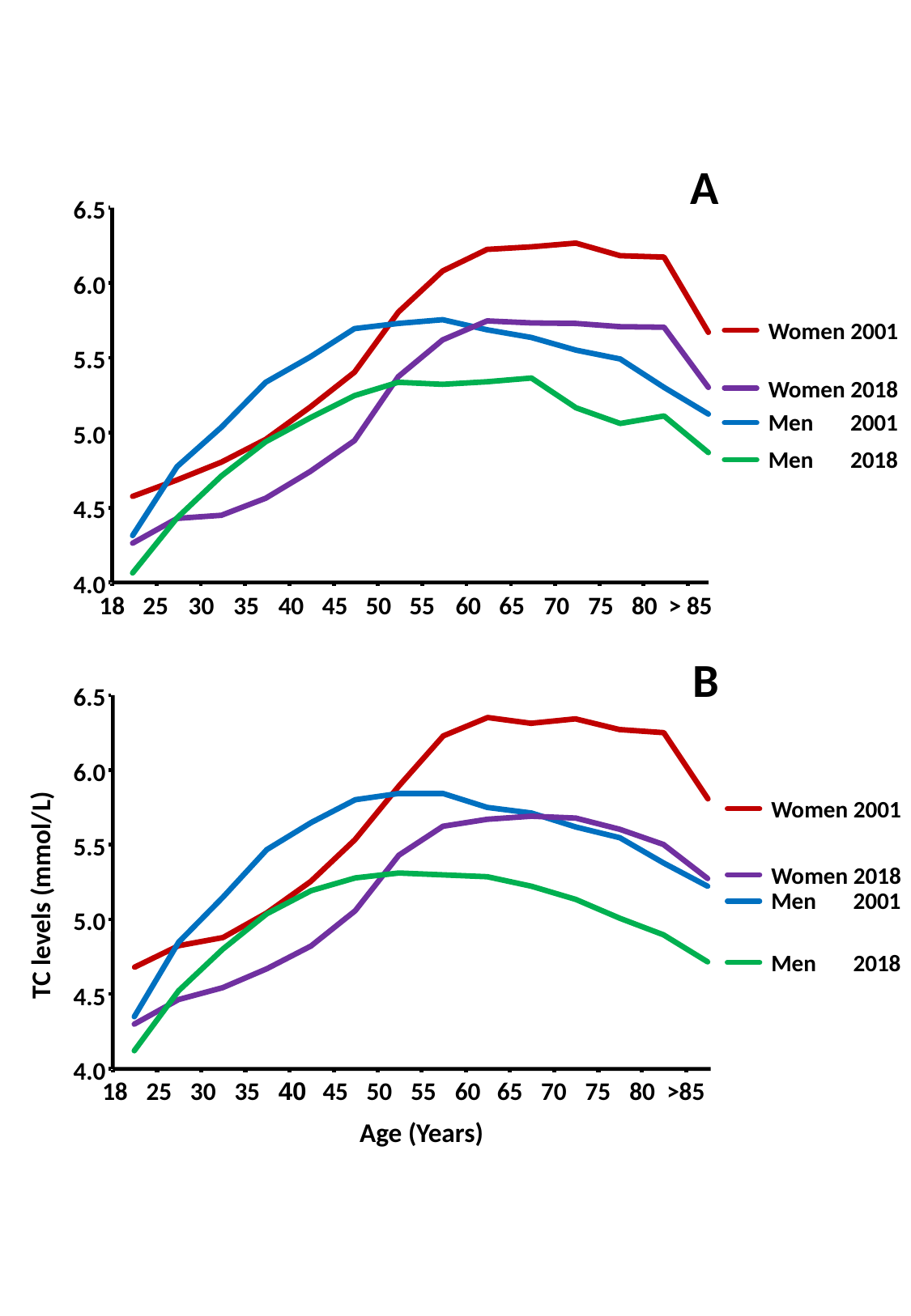

A
6.5
6.0
Women 2001
5.5
Women 2018
Men 2001
5.0
Men 2018
4.5
4.0
18
25
30
35
40
45
50
55
60
65
70
75
80
> 85
B
6.5
6.0
Women 2001
5.5
Women 2018
TC levels (mmol/L)
Men 2001
5.0
Men 2018
4.5
4.0
18
25
30
35
40
45
50
55
60
65
70
75
80
>85
Age (Years)

Supplement: Supplementary file 2 — Additional file 2: Figure 2. Sex and age related mean TC levels in 2001 and 2018 in a cohort restricting to first TC blood test ever (a), and including all blood test results regardless of multiple results per year (b). [file 12944_2021_1579_MOESM2_ESM.pptx]

## Slide 1
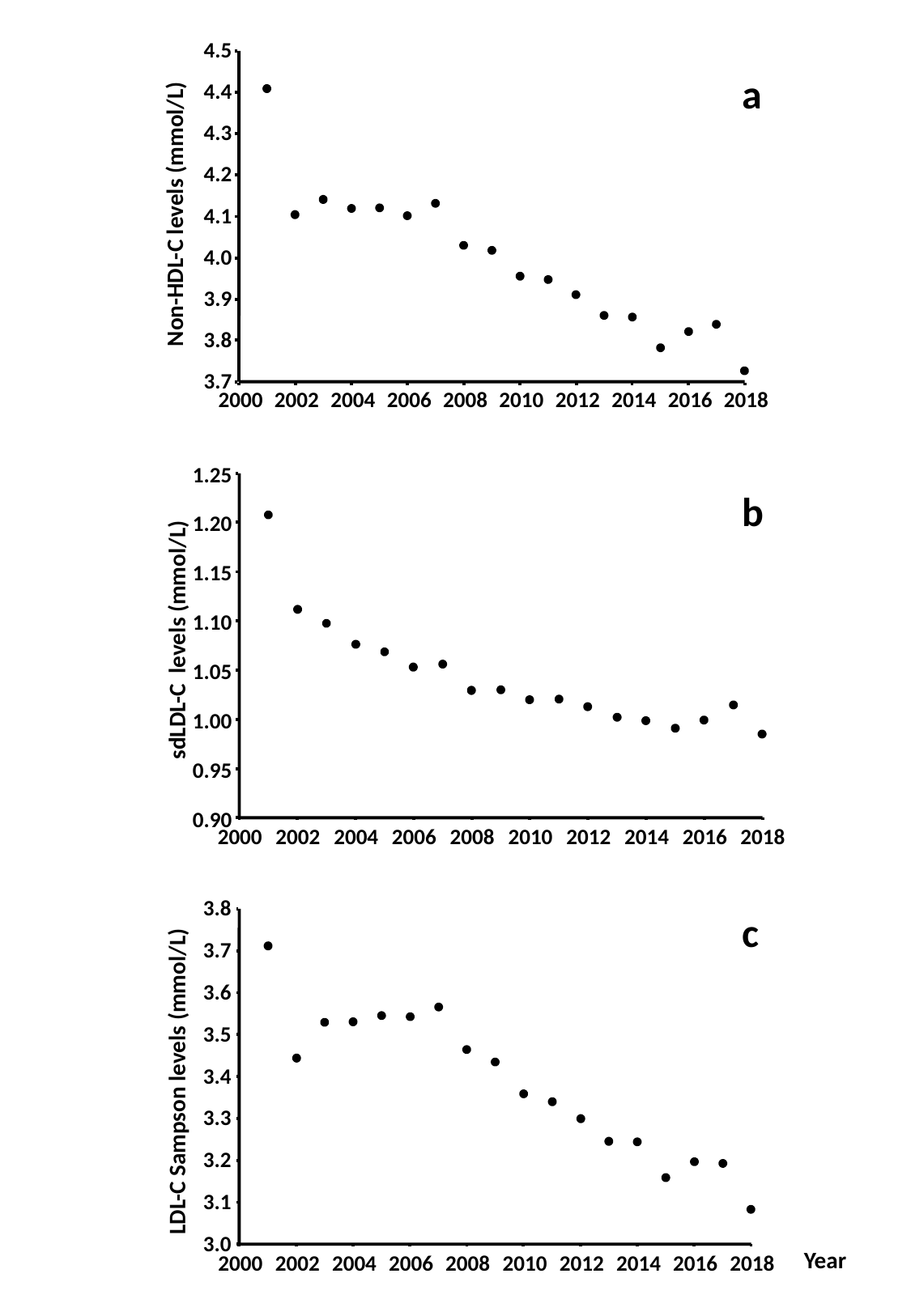

4.5
a
4.4
4.3
4.2
Non-HDL-C levels (mmol/L)
4.1
4.0
3.9
3.8
3.7
2000
2002
2004
2006
2008
2010
2012
2014
2016
2018
1.25
b
1.20
1.15
1.10
sdLDL-C levels (mmol/L)
1.05
1.00
0.95
0.90
2000
2002
2004
2006
2008
2010
2012
2014
2016
2018
3.8
c
3.7
3.6
3.5
3.4
LDL-C Sampson levels (mmol/L)
3.3
3.2
3.1
3.0
Year
2000
2002
2004
2006
2008
2010
2012
2014
2016
2018

Supplement: Supplementary file 3 — Additional file 3: Figure 3. Annual trends in Non-HDL-C (a), sdLDL-C (b) levels from 2001 to 2018, and LDL-C calculated with the Sampson equation (c). Data are presented in mean values (mmol/L). Estimates are based on the first result each year, if multiple results per patient per year were available. [file 12944_2021_1579_MOESM3_ESM.pptx]
